# Cost-Effectiveness of Pre-Exposure Prophylaxis (PrEP) in Preventing HIV-1 Infections in Rural Zambia: A Modeling Study

**DOI:** 10.1371/journal.pone.0059549

**Published:** 2013-03-18

**Authors:** Brooke E. Nichols, Charles A. B. Boucher, Janneke H. van Dijk, Phil E. Thuma, Jan L. Nouwen, Rob Baltussen, Janneke van de Wijgert, Peter M. A. Sloot, David A. M. C. van de Vijver

**Affiliations:** 1 Department of Virology, Erasmus Medical Centre, Rotterdam, The Netherlands; 2 Macha Mission Hospital and Macha Research Trust, Macha, Zambia; 3 Department of Primary and Community Care, Radboud University Nijmegen Medical Center, Nijmegen, The Netherlands; 4 Institute of Infection and Global Health, University of Liverpool, Liverpool, United Kingdom; 5 Computational Science, Faculty of Science University of Amsterdam, Amsterdam, The Netherlands; Royal Melbourne Hospital, Australia

## Abstract

**Background:**

Pre-exposure prophylaxis (PrEP) with tenofovir and emtricitabine effectively prevents new HIV infections. The optimal scenario for implementing PrEP where most infections are averted at the lowest cost is unknown. We determined the impact of different PrEP strategies on averting new infections, prevalence, drug resistance and cost-effectiveness in Macha, a rural setting in Zambia.

**Methods:**

A deterministic mathematical model of HIV transmission was constructed using data from the Macha epidemic (antenatal prevalence 7.7%). Antiretroviral therapy is started at CD4<350 cells/mm^3^. We compared the number of infections averted, cost-effectiveness, and potential emergence of drug resistance of two ends of the prioritization spectrum: prioritizing PrEP to half of the most sexually active individuals (5–15% of the total population), versus randomly putting 40–60% of the total population on PrEP.

**Results:**

Prioritizing PrEP to individuals with the highest sexual activity resulted in more infections averted than a non-prioritized strategy over ten years (31% and 23% reduction in new infections respectively), and also a lower HIV prevalence after ten years (5.7%, 6.4% respectively). The strategy was very cost-effective at $323 per quality adjusted life year gained and appeared to be both less costly and more effective than the non-prioritized strategy. The prevalence of drug resistance due to PrEP was as high as 11.6% when all assumed breakthrough infections resulted in resistance, and as low as 1.3% when 10% of breakthrough infections resulted in resistance in both our prioritized and non-prioritized scenarios.

**Conclusions:**

Even in settings with low test rates and treatment retention, the use of PrEP can still be a useful strategy in averting infections. Our model has shown that PrEP is a cost-effective strategy for reducing HIV incidence, even when adherence is suboptimal and prioritization is imperfect.

## Introduction

Despite extensive prevention efforts there were 2.6 million new HIV infections in 2009 globally [1]. While the annual number of new infections has been decreasing since 1997, there is still an urgent need for more effective prevention strategies in addition to use of condoms and behavior change. Pre-exposure prophylaxis (PrEP) with daily oral tenofovir and emtricitabine has been shown to be efficacious in preventing HIV infections [2], [3], [4]. In the recent Partner’s PrEP study among African heterosexual serodiscordant couples, daily PrEP was shown to prevent 73% of infections over three years of follow-up compared to the control arm [3]. Similarly, the TDF-2 trial among heterosexual men and women in Botswana showed that daily PrEP prevented 62% of infections over a median of 1.1 years compared to the control arm [4]. In the recent iPrEx study, daily PrEP was shown to prevent 44% of infections over a median of 1.2 years compared to the control arm in a highly sexually active cohort of men who have sex with men (MSM) [2]. The FEM-PrEP trial, among heterosexual African women did not, however, find a protective effect of PrEP, likely due to poor adherence [5].

It is unknown who should receive PrEP so that most infections are averted at the lowest cost. The cost-effectiveness of PrEP has not been established for a low-income country such as Zambia. Two hypothetical PrEP distribution scenarios could be utilized. First, PrEP could be given to more sexually active individuals, potentially by identifying a seronegative partner in a serodiscordant relationship or people with sexually transmitted infections (STIs) and their partners. Another hypothetical approach could be to randomly assign PrEP to individuals regardless of level of sexual activity in order to avert infections.

The drugs used in PrEP regimens are the same as those recommended for first-line treatment regimens. A critical issue in PrEP use is therefore the development of HIV drug resistance in the population. Potential risks associated with using the same drugs for both prevention and for treatment can be illustrated by the use of nevirapine for prevention of mother-to-child transmission [6]. Recent maternal use of nevirapine for prevention of mother-to-child-transmission was associated with a higher probability of virological failure in the mothers receiving nevirapine as part of their first-line regimen [7].

Our objective is to use mathematical modeling to explore the possibilities of daily oral PrEP optimization using realistic data collected in the rural HIV clinic at the Macha Mission Hospital in Zambia. Rural settings such as Macha often face more barriers to treatment, such as large travel distances to clinics and fewer financial resources available [8]. Particularly in these settings, optimized PrEP strategies can be of great additional value from both a public health and economic perspective. We therefore evaluated the impact of hypothetical scenarios in which PrEP is prioritized to individuals with the highest sexual activity or is distributed randomly. We could therefore determine cost-effectiveness at both ends of the PrEP distribution spectrum, from where PrEP is given to those at highest risk of becoming infected, to giving PrEP to individuals regardless of risk. We additionally aimed to evaluate the risk for resistance development.

## Methods

### Setting and Population

Our model is based on the rural population of Macha, Zambia and using data from the HIV Clinic at Macha Hospital. Macha is located in the Southern Province of Zambia, and approximately 80 km away from the nearest town, Choma [8]. The hospital serves as a district-level referral hospital for rural health centers within an 80 km radius, with 90,000 persons that are aged 12 years and over in the Macha Hospital catchment area [8]. The antenatal prevalence between 2002 [9] and 2009 [local data] was stable around 7.7%. Macha Hospital has provided care to over 7500 HIV-infected adults and children since 2005 through the Government of Zambia’s antiretroviral treatment program, with additional support from the President’s Emergency Plan for AIDS Relief (PEPFAR) through the non-governmental organization, AidsRelief [8]. Since the start of the clinic in 2005, treatment is implemented according to WHO guidelines, initially at CD4<200 cells/mm^3^, and at CD4<350 cells/mm^3^ since 2010. The HIV pharmacy is well-stocked and treatment is readily available for all diagnosed patients who drop below the treatment threshold.

### Model and Assumptions

A compartmental deterministic mathematical model was constructed and parameters were chosen to represent the Macha setting (Table 1). Our model stratifies disease progression into an acute stage, a chronic stage and two AIDS stages (Figure S1). Two AIDS stages are included because during the final months before death, patients will have limited sexual activity and are therefore assumed not to transmit HIV [10], [11]. The acute stage has a duration that ranged between 10 and 16 weeks [12]. The combined duration of the acute stage and the chronic stage is 8.5–8.7 years [10], [13]. The pre-final AIDS stage ranged between 6 and 12 months [10], [11]. Compared to the chronic stage, it was assumed that infectivity was 27–43 times higher in the acute stage [14] and 3–5 times higher in the AIDS stage [10], [11] (Table 1).

**Table 1:** Model Parameters.

Description	Estimate or Range*		Reference
Test rate	10–20%		Macha, Zambia
* Rate of being tested in the acute stage of HIV*	50% of the test rate		Assumption**
* Rate of being tested in the chronic stage of HIV*	test rate		Macha, Zambia
* Rate of being tested in the AIDS stage*	test rate +10%		Macha, Zambia
Disease stages duration			[10], [11], [12], [13]
* Acute stage*	10–16 weeks		
* Chronic stage*	8.31–8.43 years		
* AIDS stage*	6–12 months		
* Final AIDS stage*	7–13 months		
Proportion of people in sexual risk groups			Model Calibration
* Highest* ***	1.0%–2.9%		
* 2^nd^* ***	15.1%–24.0%		
* 3^rd^*	10%		
* Lowest*	63.1%–73.9%		
Number of partners per year in each sexual risk group			Model Calibration
* Highest* ***	7–31		
* 2^nd^* ***	1.5–2.6		
* 3^rd^*	0.1		
* Lowest*	0.03		
Mortality rates per year			[39]
* Population*	0.02		
* Chronic HIV stage*	0.098		
* AIDS stage*	0.63		
* On treatment during chronic stage, first 3 months*	0.05–0.098		
* On treatment during chronic stage, second 3 months*	0.03–0.06		
* On treatment during chronic stage, 6+ month*	0.02–0.05		
* On treatment during AIDS stage, first 3 months*	0.1–0.3		
* On treatment during AIDS stage, second 3 months*	0.05–0.12		
* On treatment during AIDS stage, 6+ month*	0.03–0.06		
Linkage to care from test to treat	70%		Macha, Zambia
Proportion of people on PrEP			
* Non-prioritized PrEP*	40–60%†		Assumption
* Prioritized PrEP (approximately half of highest two sexual risk groups)*	5–15%‡		Assumption
Effectiveness of PrEP			[2], [3], [4]
* Moderate Adherence*	20–60%		
* High Adherence*	50–90%		
Reduction in transmissibility of those patients on treatment	90–100%		[25], [26], [27]
Rate of resistance among those infected despite use of PrEP	10%, 50%, 100%		Assumption
Rate of discontinuation of PrEP (not due to resistance)	4–5%		[40]
Number of HIV tests per year on PrEP	1–4		Assumption
Number of HIV clinic visits in first year	8		Macha, Zambia
Number of yearly HIV clinic visits after first year	4		Macha, Zambia
**Costs**			
Cost of PrEP per year (TDF/FTC) (§)	$126 ($137.12)	[28], [29]	
Cost of testing negative for HIV per test (§)	$1 ($3.78)	Macha, Zambia, [28]	
Cost of testing positive for HIV per test (§)	$3.84 ($9.4)	Macha, Zambia, [28]	
Cost of an inpatient day in the hospital	$10.27	[28]	
Cost of an outpatient visit in the hospital	$2.78	[28]	
Cost of treatment per year (TDF/FTC+EFV) (§)	$194 ($243)	[29]	
Cost of a CD4 Count test (§)	$31–$39($34–$42)	Macha, Zambia, [28]	
Cost discounting rate per year	3%		
Exchange rate, Zambian Kwacha to USD over year 2011	3845∶1		

* All ranges are uniformly distributed, except where indicated.

** Due to window phase of antibody-based test.

*** Not uniformly distributed, see figure S2.

† Not uniformly distributed, median 43% over 10 years;

‡ Not uniformly distributed, median 12% over 10 years;

§ Comprehensive costs, including costs of outpatient visits, additional laboratory tests, laboratory personnel.

Individuals that test positive for HIV can reduce their risk behavior [15], [16], [17], largely due to a reduction in acquisition of new partners [15]. Based on recent work done in neighboring Zimbabwe, it is assumed in our model that patients will reduce the acquisition of new partners by 0–40% [18].

#### Model Description and Validation

Following earlier model’s methods for defining risk structure [19], [20], the model identifies four sexual activity groups ranging in the number of new sexual partners per year [21]. Data about the proportion of individuals in a particular sexual activity group and their number of new partners are not available. Using the Monte Carlo filtering techniques [22] we parameterized the different sexual activity groups and only accepted the 1795 simulations that were associated with a prevalence of 7.7% (±0.05%) from 2002 until 2009 in accordance with Macha. Monte Carlo filtering allowed us to test the impact of PrEP over a wide range of sexual activities, as a wide variety of sexual risk group combinations resulted in the appropriate HIV prevalence (Table 1).

In summary, the highest sexual activity group had an average of 13 new partners per year and made up on average just 2% of the population, representing a core group of highly sexually active individuals. This group is instrumental in determining the peak of the epidemic. Only simulations where this group was small and their number of partners were high allowed the epidemic to peak appropriately. The second highest sexual activity group had on average 2 new partners per year and made up a more substantial 18% of the population, representing individuals whom are not in steady or monogamous relationships. This is the group is an important factor in determining where the equilibrium of the epidemic is reached. The only simulations that were accepted into the analysis were the ones in which this group allowed the epidemic to reach an equilibrium prevalence of 7.7% (±0.05%) from 2002–2009 in accordance with Macha data. The two lowest groups had <1 new sexual partner per year, representing individuals in long term relationships or marriages. The final distribution of proportion of sexual activity groups and number of new partners per year are given in Figure S2. Other variables used to calibrate the model included: transmissibility during the acute stage of infection, transmissibility during the AIDS stage of infection, the rate at which individuals moved from acute to chronic infection, rate at which individuals move from the AIDS stage to the AIDS final stage, and the rate of mixing between sexual risk groups (epsilon). Full model description including equations can be found in the Text S1.

#### HIV testing

Approximately 10% of individuals aged 12 and older undergo an HIV-test yearly in Macha. In our model, we studied the impact on the HIV-epidemic of test rates that were ranged randomly between the current level and a double proportion of 20% [23]. We assumed different test rates for different stages of disease progression (Table 1).

#### Treatment

After a positive HIV-test, 70% of individuals are retained in care. Treatment is then started at CD4<350 cells/mm^3^. In the AIDS stage, there is therefore immediate treatment after diagnosis. Additionally it takes approximately 4 years to progress from infection to CD4<350 cells/mm^3^ [24]. Treatment reduces the infectivity by 90–100% as compared to the chronic stage [25], [26], [27].

### Scenario Assumptions

#### Baseline

Our baseline in this model is the current practice in Macha (i.e. test rate 10–20%, retention 70% and start of treatment at CD4<350 cells/mm^3^).

#### Non-Prioritized versus Prioritized PrEP distribution

We examined the impact of two hypothetical scenarios where PrEP is perfectly and imperfectly prioritized to represent both ends of the prioritization spectrum. In the first hypothetical scenario, we examined the impact of completely perfect prioritization by assigning approximately half of the individuals in the two highest sexual activity groups, 5–15% of the population (4,500–13,500 individuals), to receive PrEP. We assigned just half of the highest sexual activity groups, as identifying those groups completely would likely not be feasible. In the second hypothetical scenario where PrEP is imperfectly prioritized, PrEP is assigned to half of the population in a non-prioritized manner by assigning PrEP to 40–60% of the population at random (36,000–54,000 individuals). Time to reach PrEP coverage was 1–2 years.

#### PrEP adherence

Adherence is key in PrEP use as illustrated by all recent PrEP studies [2], [3], [4], [5]. Since it is unknown what level of adherence would be expected in Macha, we examined a high population-level adherence scenario and ranged PrEP effectiveness from 50%–90%, derived from the highly adherent in recent PrEP trials [2], [3], [4], and a moderate population-level PrEP adherence scenario, where effectiveness ranged from 20%–60%.

#### Drug resistance

Rates of drug resistance due to PrEP are currently unknown. Drug resistance may emerge in individuals who become infected with HIV despite the use of PrEP. It is unknown how rapidly resistance will emerge after PrEP failure. We therefore evaluated a scenario with low resistance development, where resistance develops in 10% of breakthrough infections (infections despite the use of PrEP). We also evaluated a moderate resistance and high resistance scenario, where resistance emerges in 50% and 100% of breakthrough infections respectively. The prevalence of drug resistance is expressed as the proportion of individuals with a resistant virus over the total number of infections in the population.

### Cost-effectiveness Analysis

In order to evaluate the feasibility of the range in PrEP implementations, we conducted a cost-effectiveness analysis. Each compartment in our deterministic model was assigned a range of cost and quality adjusted life year (QALY) depending on the intervention (Table 1, and Tables S1, S2, S3). A QALY of 1 means one year of life lived in perfect health. As our base, a susceptible person not on PrEP was considered to have no reduction in health-related quality of life. Rates of HIV clinical tests were taken from Macha’s standard practice, including the different types of tests and how frequently they are administered. Costs and rates for hospitalization of HIV infected persons, opportunistic infections (Table S4), HIV testing, and treatment, were all taken into account using costs from Macha and the WHO-CHOICE costing database [28]. Current ARV costs were taken from the 2011 Clinton Health Access Initiative negotiated prices [29]. An intervention is said to be cost-effective if it costs less than three times the gross national income (GNI) per capita ($3210 in Zambia [30]) per QALY gained. An intervention is defined as very cost-effective at a cost up to one times the GNI per capital ($1070 in Zambia [30]) per QALY [31], [32]. We calculated both the average cost-effectiveness ratios where we compared each scenario to baseline, and the incremental cost-effectiveness ratios where we compared each scenario to the next least-costly scenario [33]. We follow methodological guidelines on cost-effectiveness analysis [33], and only consider the latter as meaningful for making optimal resource allocation decisions. All costs have been discounted yearly (converting future costs into present terms) at the standard of 3%.

### Sensitivity Analysis

We performed one-way deterministic sensitivity analysis of cost-effectiveness where our baseline model for comparison was the prioritized PrEP model with moderate PrEP adherence. Eight key input variables, HIV prevalence, PrEP efficacy, proportion of people in highest two sexual activity groups on PrEP, number of HIV tests per year for those on PrEP, cost of antiretroviral drugs, total costs depending on the exchange rate, cost and QALY discounting were considered to identify the sensitivity of our model. We also determined the amount of additional money that could be spent on infrastructure and programmatic costs of implementing prioritized PrEP and have the intervention still be (very) cost-effective.

### Ethics Statement

Written informed consent was obtained from the study participants. Ethical approval was granted by the University of Zambia Biomedical Research Ethical Committee in 2008 before data collection began.

## Results

### Baseline Scenario: Start of Treatment at CD4<350 Cells/mm^3^

The impact of treatment alone under the current guidelines of treatment at CD4<350 cells/mm^3^ reduces incidence, showing an 18% decline in new infections over 10 years. The prevalence remained stable at 7.7% after 10 years, as treatment dramatically reduces mortality and patients therefore remain alive.

### Prioritized Versus Non-Prioritized PrEP

**Figure 1:**
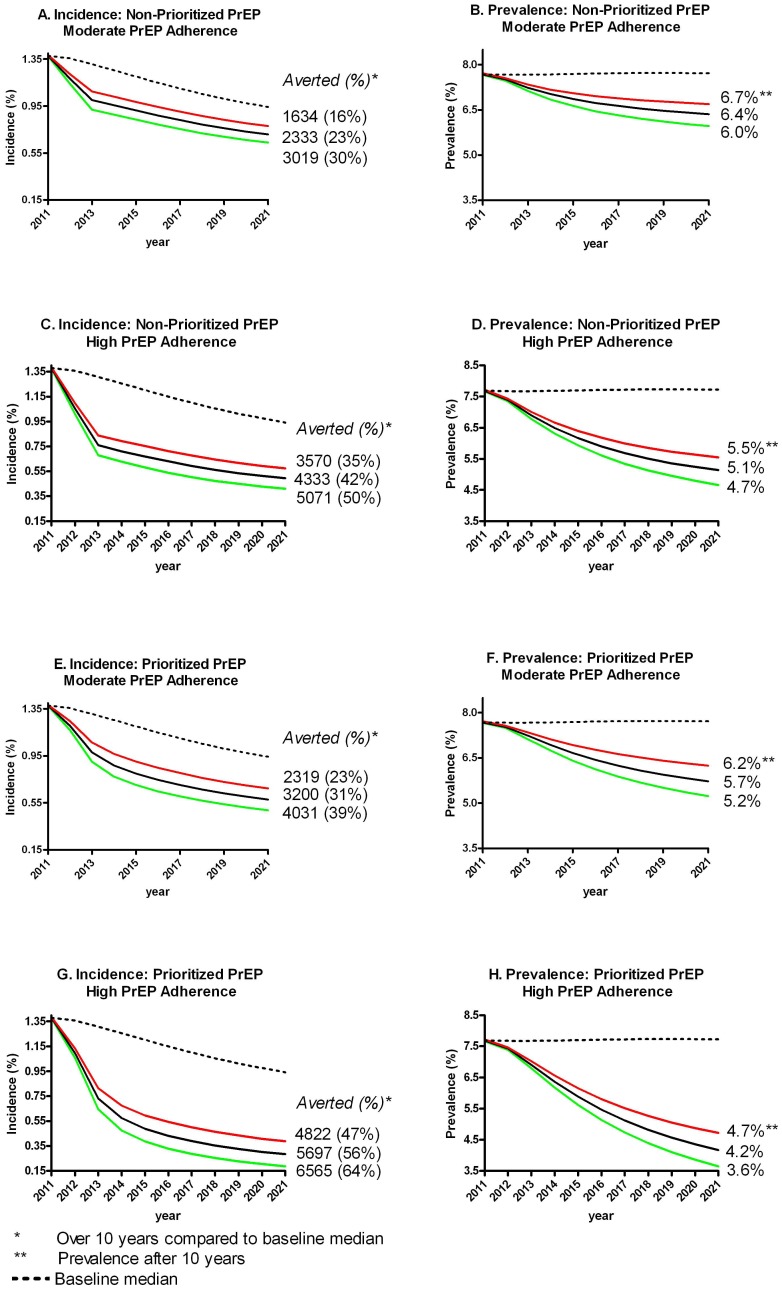
Prioritizing highest sexual risk groups versus a non-prioritized PrEP strategy, incidence and prevalence.

Compared to our baseline scenario of starting treatment at CD4<350 cells/mm^3^, prioritizing PrEP will result in 3200 infections averted over 10 years (31% reduction; interquartile range (IQR) 23%–39%), whereas a non-prioritized PrEP strategy will result in just 2333 infections averted (23% reduction; IQR: 16–30%) (Figure 1A, 1E). The prevalence in the prioritized approach is lower after 10 years, at 5.7% (IQR: 5.2%–6.2%), compared to a prevalence of 6.4% (IQR: 6.0%–6.7%) in the non-prioritized strategy (Figure 1B, 1F).

### Impact of Adherence

As expected, high PrEP adherence had a strong impact on the HIV epidemic as compared to moderate PrEP adherence in both the prioritized and non-prioritized strategies. The impact, however, was stronger than expected. In the non-prioritized strategy, compared to baseline, an estimated 4333 infections (42% reduction; IQR: 35%–50%) were averted with high adherence to PrEP (Figure 1C), 2000 more than with moderate adherence. In the prioritized strategy, compared to baseline, an estimated 5697 infections (56% reduction; IQR: 47%–64%) were averted with high adherence to PrEP (Figure 1G), almost 2500 more than with moderate adherence. High adherence also has a strong impact on the HIV prevalence after 10 years of the intervention, with a median prevalence of 5.1% (IQR: 4.7%–5.5%) in the non-prioritized strategy and 4.2% (IQR: 3.6%–4.7%) in the prioritized strategy (Figure 1D, 1H).

### Drug Resistance and PrEP

**Figure 2:**
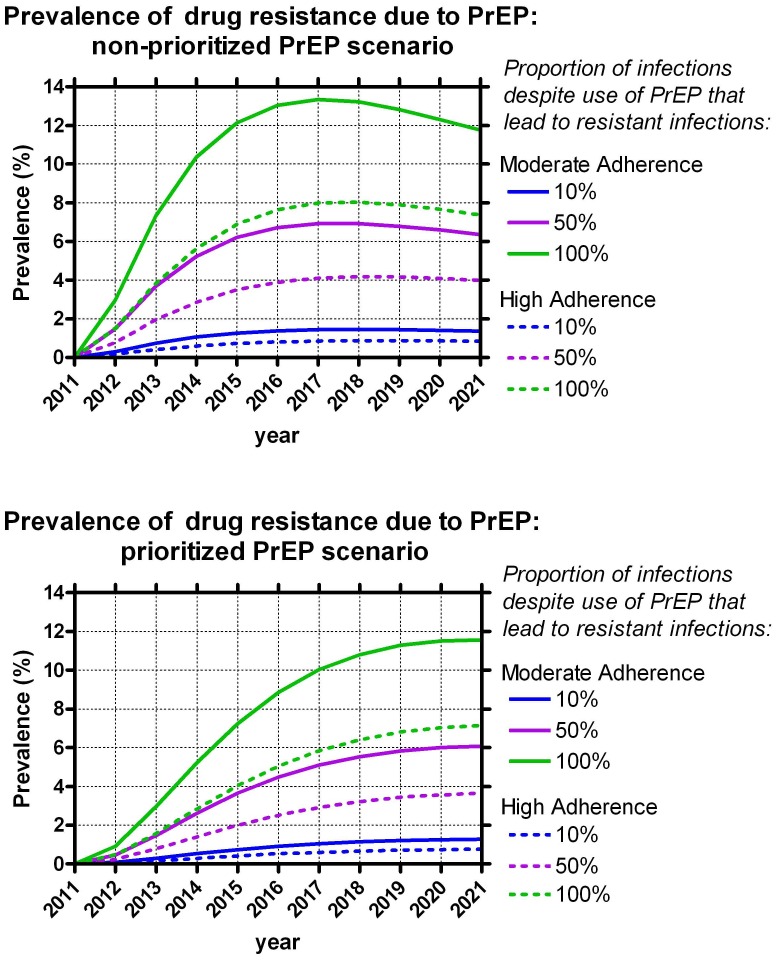
Prevalence of drug resistance due to PrEP over 10 years.

Investigating the impact of PrEP on resistance development showed that when 100% of breakthrough infections developed a drug resistant virus with moderate adherence, the prevalence of drug resistance due to PrEP was strikingly high. In the prioritized PrEP scenario, there was an 11.6% (IQR 10.3%–12.8%) prevalence of drug resistance due to PrEP alone after 10 years (Figure 2). Assuming a 50% and 10% drug resistance rate among PrEP users resulted in a 6.1% (IQR 5.3%–6.8%) and 1.3% (IQR 1.1%–1.4%) drug resistance prevalence due to PrEP after 10 years. The results were almost identical in our non-prioritized scenario.

Adherence, however, appears to strongly impact the prevalence of drug resistance due to PrEP. With high adherence, the drug resistance due to PrEP was 7.1% (IQR 5.3%–8.8%) in the prioritized scenario, approximately 4% lower than in the moderate adherence scenario, assuming a 100% drug resistance rate among PrEP users. Assuming a 50% and 10% drug resistance rate among PrEP users resulted in a 3.7% (IQR 2.6%–4.6%) and 0.8% (IQR 0.5%–1.0%) drug resistance prevalence due to PrEP after 10 years in the prioritized scenario. The results were again almost identical in our non-prioritized scenario with high adherence.

### Cost-effectiveness

We evaluated the cost-effectiveness of the prioritized and non-prioritized PrEP interventions compared with the baseline (Table 2). Our baseline scenario cost $4.3 million (IQR: $3.8–$4.7 million) over 10 years. Of that amount, approximately 54% would be covered under PEPFAR as long as PEPFAR continues. A total of 10222 infections would be expected over 10 years.

**Table 2:** Cost-effectiveness of PrEP interventions, and additional money available for programmatic costs in each intervention over 10 years for the intervention to remain very cost-effective, or cost-effective.

		*Total Effects*					*Amount that can be spent and still have the intervention be:*	
Intervention	Total cost in $ Millions (*) (IQR**)	Infections averted (% averted) (IQR)	QALYs gained (IQR)	Average Cost- Effectiveness Ratio	Incremental Cost- EffectivenessRatio†	Conclusion	Very Cost-Effectivein $ Millions (IQR)	Cost-Effective in $ Millions (IQR)
Baseline, standard care, no PrEP	4.3 (54%) *(3.8, 4.7)*	−	−	−	−	−	−	−
Non-prioritized PrEP, PrEPrandomly distributed	48.2 (4%) *(45.7, 50.3)*	2333 (23%)*(16%, 30%)*	23571 (15680, 31764)	$1843 ($1386, $2724)	Dominated‡	−	−	−
Prioritized PrEP to most sexually active	15.8 (13%) *(14.7, 16.9)*	3200 (31%)*(23%, 39%)*	36216 (26174, 45690)	$323 ($257, $428)	$323 ($257, $428)	Very Cost-Effective	25.2 (16.2, 33.2)	98.4 (69.4, 124.9)

* Percentage of total costs that are currently covered under PEPFAR –primarily ARV treatment.

** IQR: Interquartile range.

† When non-prioritized PrEP is compared to prioritized PrEP.

‡ Less effective and more costly than prioritized PrEP.

The prioritized PrEP strategy cost an additional $11.5 million (IQR: $11.1–$13.4 million) compared to the baseline strategy. A median of 36,216 QALYs would be gained (IQR: 26,174, 45,690) with the prioritized scenario over 10 years.

The non-prioritized PrEP strategy cost an additional $43.9 million (IQR: $41.4, $46.0 million) compared to baseline. A median of 23,571 QALYs would be gained (IQR: 15,680, 31,764) with the non-prioritized scenario over 10 years.

Based on the interpretation of average cost-effectiveness ratios only, both strategies can be considered (very) cost-effective. However, the interpretation of incremental costs and effects of the prioritized PrEP strategy as compared to the non-prioritized strategy reveals that the former strategy is both less costly and more effective, and ‘dominates’ the latter. This means that the non-prioritized PrEP strategy cannot be considered economically attractive. The incremental cost-effectiveness ratio of the prioritized PrEP strategy is $323 per QALY (IQR: $257, $428) and this strategy can thus be considered very cost-effective.

### Sensitivity Analysis

**Figure 3:**
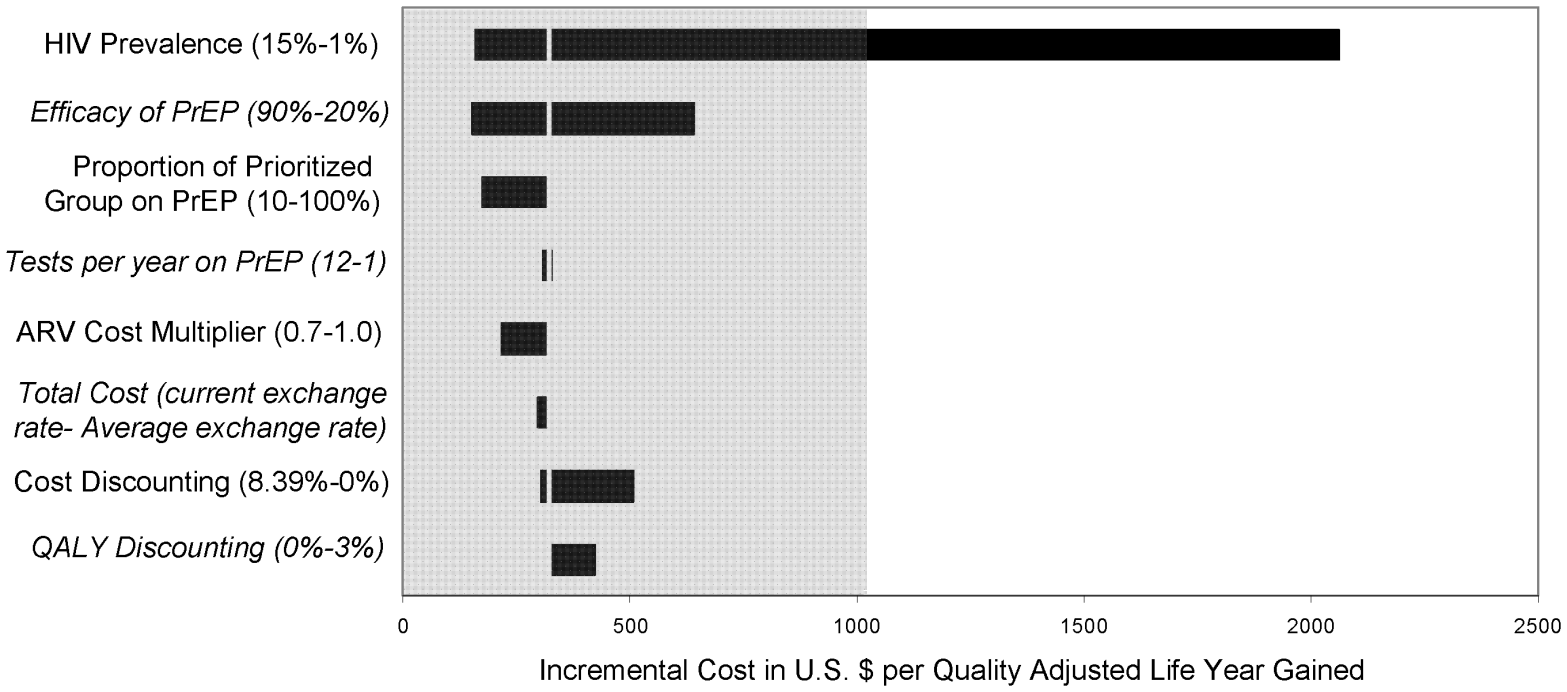
One-way sensitivity analyses of the incremental cost-effectiveness of PrEP.

One-way sensitivity analyses (Figure 3) highlighted the eight key input parameters of our model. Even when just 10% of the highest two sexual activity groups are prioritized for PrEP (2% of the total population, or 1,800 individuals), the cost per QALY is actually lower than when approximately half of the two highest sexual activity groups are prioritized, at only $177 per QALY. This shows that targeting just a small fraction of those individuals in a higher sexual activity group would be optimal from a cost-effectiveness perspective.

It appears that PrEP will be more cost-effective in regions with higher HIV prevalence at $161 per QALY in a region with a prevalence of 15%. In contrast, prioritized PrEP is no longer very cost-effective for a prevalence of 1%, at $2062 per QALY. The remainder of the parameters–frequency of HIV testing on PrEP, PrEP effectiveness (as controlled by adherence [2]), cost of ARVs, cost and QALY discounting rate, and exchange rate– did not result in large differences in cost-effectiveness from our baseline prioritized model.

If implemented, the prioritized PrEP strategy could spend an additional $25.2 million over 10 years on infrastructure and programmatic costs and remain very cost-effective ($94.8 million to remain cost-effective) (Table 2).

## Discussion

Our model has shown that PrEP is a cost-effective strategy for reducing HIV incidence, even when prioritized imperfectly and distributed regardless of risk of acquiring HIV. If PrEP can be perfectly prioritized to the most sexually active individuals, it is a very cost-effective prevention method and averts 31% of infections averted over 10 years at $323 per QALY. Even when prioritizing just a small fraction of the highly sexually active, PrEP is very cost-effective at $177 per QALY gained.

The prevalence of drug resistance due to PrEP could be high. It is therefore important to closely monitor patients who become infected despite the use of PrEP for resistance. Drug resistance is, however, much lower when adherence to PrEP is higher.

A strength of our study is access to cost and epidemiologic data from Macha, a rural setting in Zambia. Access to this dataset enables us to make reliable predictions about the potential implementation of PrEP. Another strength is that there is limited migration into and out of Macha as transportation and mobility are limited. Migration can have a major impact on a local HIV epidemic, and also on a mathematical model attempting to capture HIV dynamics in a population. The population in Macha has, however, remained fairly stable over time.

A limitation of our modeling approach is that highly sexually active individuals are difficult to identify. Nonetheless, we found that cost-effectiveness remained the same if only 10% of the high sexual activity groups could be prioritized (2% of the total population). Health care providers could begin with prioritizing those individuals who present with STI symptoms at clinics, or are identified as the seronegative partner in a serodiscordant relationship. Over a wide spectrum of adherence and PrEP prioritization, we predict that PrEP will reduce HIV incidence and will be cost-effective.

Our model does not take into account administrative program costs [34], as they would vary widely depending on the precise intervention used. We have also not included indirect costs, as these are very difficult to quantify. We have instead shown the additional amount that could be spent on those costs and retain cost-effectiveness. The government of Zambia or donors could invest an additional $25,200,000 over 10 years in the implementation of prioritized PrEP, and have it remain very cost-effective.

Previous models have shown the potential impact of PrEP. A model by Pretorius *et al.* evaluated cost-effectiveness in a generalized South African epidemic [35]. When all individuals were assigned to receive PrEP, they showed a decrease in incidence in 2025 of about 40% compared to their baseline. This is approximately in line with our findings, albeit a bit low considering that we assigned PrEP to half of our population.

A model by Abbas *et al*. investigated the factors influencing the emergence and spread of HIV drug resistance arising from PrEP rollout, based on a general mature epidemic in sub-Saharan Africa [36]. In their PrEP scenario analyses, the largest decrease in infections was achieved with a non-prioritized strategy (31% in an optimistic scenario, similar to our “high adherence” scenario; 7% in realistic, similar to our “moderate adherence”) and the smallest decrease with the prioritized-by-activity strategy (8% in optimistic, 3% in realistic). The benefits of PrEP in this model were much lower than estimates from our model. Reasons for this could be their definitions of optimistic and realistic, as well as the level of protection offered from PrEP.

In iPrEx, HIV drug resistance due to PrEP was not a major issue [2], likely due to monthly monitoring of participants for seroconversion. The only resistance found was in those with a false negative HIV test at randomization and started PrEP. The study by Abbas *et al.* has also examined the emergence of drug resistance due to PrEP in a heterosexual sub-Saharan epidemic [36]. In agreement with our results, the Abbas model has shown that there is not much difference in the prevalence of drug resistance in a non-prioritized or prioritized PrEP scenario, but that higher PrEP adherence would result in less drug resistance. The total prevalence of resistance in their optimistic scenario was about 1.9–2.5% and 9.2–9.9% in their realistic scenario. If we had evaluated the same measure of drug resistance, these figures are likely lower than ours.

Several prevention strategies using antiretroviral drugs have been shown to be effective in reducing new infections with HIV. These strategies include antiretrovirals for prevention of mother-to-child transmission [7], [37], topical tenofovir as an intra-vaginally applied microbicide [38] and earlier start of treatment as prevention [27]. Our baseline model looks at the impact of starting treatment at a CD4 count of <350 cells/mm^3^, and found that starting treatment at that cutoff is already an intervention. Incidence was reduced by more than 30% after 10 years.

iPrEx is the first study to be published looking at the efficacy of PrEP, and was investigating an MSM community with high numbers of sexual contacts. Results on the effectiveness of PrEP in heterosexuals have also been reported [3], [4], [5]. FEM-PrEP trial had enrolled 1,951 African women to investigate the efficacy of TDF/FTC as PrEP, and was recently discontinued due to lack of an effect, likely due to adherence [5]. Two studies, however, found more encouraging results. The Partner’s PrEP study of 4,758 serodiscordant couples based in Kenya and Uganda found a 73% reduction in risk of the participants on TDF/FTC compared to placebo [3]. Similarly, the CDC’s Botswana-based TDF2 study found a 63% reduction in risk of those assigned to receive daily PrEP [4]. Adherence to PrEP is key as the highly adherent in both iPrEx and Partner’s PrEP appeared to have the same level of high PrEP efficacy, showing that PrEP works similarly irrespective of MSM or heterosexual transmission.

Even in settings with low test rates and treatment retention, the use of PrEP can still be a useful strategy in averting infections. Our model has shown that PrEP is a cost-effective strategy for reducing HIV incidence, even when adherence is suboptimal and prioritization is imperfect. Particularly in high prevalence settings, prioritizing PrEP to high sexual activity groups could be a cost-effective way to curb the epidemic. Effective ways to prioritize high sexual activity groups in a heterosexual epidemic and maximize adherence should be investigated further in order to increase the numbers of infections averted and cost-effectiveness.

## Supporting Information

Figure S1 **Structure of the compartmental deterministic model.**
(DOC)

Figure S2 **Distributions of sexual activity groups.**
(TIF)

Table S1 **Table with assumed utility weightings for QALYs.**
(DOC)

Table S2 **Table with costs used in treating opportunistic infections, per unit.**
(DOC)

Table S3 **Table with costs used in diagnosing opportunistic infections and monitoring HIV, per test.**
(DOC)

Table S4 **Table of opportunistic infection rates, hospitalization & treatment assumptions.**
(DOC)

Text S1 **Model Description and equations.**
(DOC)
