# Supplementary material for: Cost-Effectiveness of Pre-Exposure Prophylaxis (PrEP) in Preventing HIV-1 Infections in Rural Zambia: A Modeling Study
Source: PLoS One. 2013 Mar 18;8(3):e59549. doi: 10.1371/journal.pone.0059549 (PMC3601101; doi:10.1371/journal.pone.0059549)
Supplement: Figure S1 — Structure of the compartmental deterministic model. (DOC) [file pone.0059549.s001.doc]

**Figure S1: Structure of the compartmental deterministic model**, full description on next page**:**


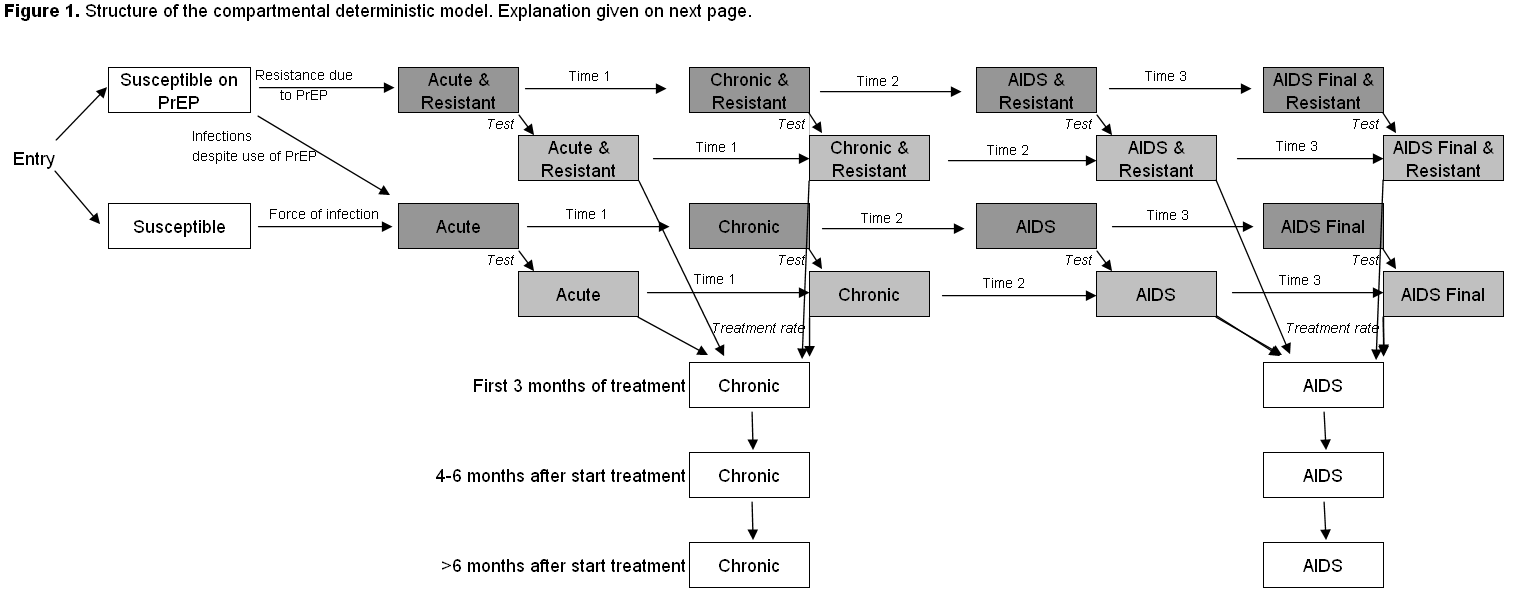


**1:** *Structure of the model, continued*. The figure is a schematic representation of the dynamic process by which individuals become infected with HIV or resistant HIV due to PrEP, become tested and are given treatment. A proportion of individuals on PrEP will develop a drug resistant virus due to PrEP use, and will progress through infection with a resistant virus. The force of infection is the rate by which susceptible individuals, on PrEP or not, become infected. Without treatment, infected patients progress through four stages: the acute stage, the chronic stage, an AIDS stage in which patients have a limited level of sexual activity and the final AIDS stage in which patients have no sexual activity. Individuals in the dark grey boxes have not been diagnosed with HIV and did not adapt their risk behavior. After testing positive for HIV, individuals move to a light grey box with the corresponding disease stage and adapt their behavior. Individuals on treatment (white boxes on bottom) move through three different periods defined by the time since start of antiretroviral drug therapy and the disease stage in which treatment was started. These periods and disease stages were included as mortality depends on time since start of treatment and the CD4 count at start of treatment.

All boxes have different mortalities. All boxes (except the ones with susceptible individuals) contribute to the force of infection, but all with different infectivity. The mathematical equations are listed in Text S1. Parameters and ranges can be found in Table 1, as well as in Tables S1-S4.
