# Supplementary material for: Cost-Effectiveness of Pre-Exposure Prophylaxis (PrEP) in Preventing HIV-1 Infections in Rural Zambia: A Modeling Study
Source: PLoS One. 2013 Mar 18;8(3):e59549. doi: 10.1371/journal.pone.0059549 (PMC3601101; doi:10.1371/journal.pone.0059549)
Supplement: Table S1 — Table with assumed utility weightings for QALYs. (DOC) [file pone.0059549.s003.doc]

| **Table S1:** Assumed utility weightings for QALYs | |
| --- | --- |
| **Status** | **Utility Weight*** |
| Susceptible | 1.0 |
| Susceptible on PrEP | 98-100%: 1.0  0-2%: 0.9-1.0** |
| Acutely infected | 0.94 |
| Chronically infected | 0.94 |
| Infected early AIDS stage | 0.82 |
| Infected late AIDS stage | 0.7 |
| Infected on treatment | 0.94 |
| *Weights based on a pooled analysis by Tengs and Lin (2002) [1] | |
| **0-2% will suffer from renal failure on these ARVs[2], which could result in a reduction in quality of life, or go unnoticed. | |

1. Tengs TO, Lin TH (2002) A meta-analysis of utility estimates for HIV/AIDS. Med Decis Making 22: 475-481.

2. Bendavid E, Grant P, Talbot A, Owens DK, Zolopa A (2011) Cost-effectiveness of antiretroviral regimens in the World Health Organization's treatment guidelines: a South African analysis. AIDS 25: 211-220.
