# Supplementary material for: Cost-Effectiveness of Pre-Exposure Prophylaxis (PrEP) in Preventing HIV-1 Infections in Rural Zambia: A Modeling Study
Source: PLoS One. 2013 Mar 18;8(3):e59549. doi: 10.1371/journal.pone.0059549 (PMC3601101; doi:10.1371/journal.pone.0059549)
Supplement: Table S2 — Table with costs used in treating opportunistic infections, per unit. (DOC) [file pone.0059549.s004.doc]

| **Table S2:** Costs used in treating opportunistic infections, per unit | | | |
| --- | --- | --- | --- |
| **Drug** | **Unit** | **Cost, USD** | **Source** |
| Aciclovir | 200mg | $0.014 | Macha, Zambia |
| Amoxicillin | 250mg | $0.037 | Macha, Zambia |
| Amphotericin B | 50mg | $6.12 | Macha, Zambia |
| Ciprofloxacin | 250mg | $0.029 | Macha, Zambia |
| Doxycycline | 100mg | $0.011 | Macha, Zambia |
| Fluconazole | 200mg | $0.125 | Macha, Zambia |
| RHE | 150/75/400mg | $0.025 | [1] |
| RHZE | 150/75/400/275mg | $0.06 | [1] |

1. Stop TB Partnership (2011) Global Drug Facility Product Catalogue. Geneva: Global Drug Facility.
