# Supplementary material for: Cost-Effectiveness of Pre-Exposure Prophylaxis (PrEP) in Preventing HIV-1 Infections in Rural Zambia: A Modeling Study
Source: PLoS One. 2013 Mar 18;8(3):e59549. doi: 10.1371/journal.pone.0059549 (PMC3601101; doi:10.1371/journal.pone.0059549)
Supplement: Table S3 — Table with costs used in diagnosing opportunistic infections and monitoring HIV, per test. (DOC) [file pone.0059549.s005.doc]

| **Table S3:** Costs used in diagnosing opportunistic infections and monitoring HIV, per test | |
| --- | --- |
| **Test/Supply** | **Cost, USD*** |
| Antigen test | $0.82 |
| CD4 Test | $31-$39 |
| Chest X-ray film | $0.83 |
| Creatinine test | $0.15 |
| Hepatitis B | $1.87 |
| Lumbar puncture | $2.78 |
| Microscope slide | $0.03 |
| RPR | $0.16 |
| Cost of lab personnel, per minute | $0.007 |
| *All costs taken from Macha, Zambia | |
