# Supplementary material for: Cost-Effectiveness of Pre-Exposure Prophylaxis (PrEP) in Preventing HIV-1 Infections in Rural Zambia: A Modeling Study
Source: PLoS One. 2013 Mar 18;8(3):e59549. doi: 10.1371/journal.pone.0059549 (PMC3601101; doi:10.1371/journal.pone.0059549)
Supplement: Table S4 — Table of opportunistic infection rates, hospitalization & treatment assumptions. (DOC) [file pone.0059549.s006.doc]

| **Table S4:** Opportunistic infection rates* and hospitalization & treatment assumptions** | | | | | | | |
| --- | --- | --- | --- | --- | --- | --- | --- |
| **Opportunistic Infections (OIs), observed in Macha dataset** | **Rate of OI** | | | **Percent Hospitalized** | **Duration of Hospitalization** | **Drugs used to treat disease:** | **Additional lab tests needed** |
|  | **Chronic** | **AIDS** | **On Treatment** |  |  |  |  |
| Herpes Zoster | 2.00% | 6.40% | 4.10% | 5-15% | 7 days | Aciclovir, 5x800mg, 7 days | - |
| Diarrhea | 0.46% | 3.10% | 2.10% | 5-10% | 3-7 days | Ciprofloxacin, 2x500mg 3-5 days | - |
| Tuberculosis | 0.92% | 3.10% | 2.20% | 90-100% | 14 days | RHZE 2 months, RHZ 4 months | Chest X-ray, 3 acid-fast bacillus (AFB) smears |
| Pneumonia | 1.00% | 2.20% | 1.20% | 75-85% | 5-7 days | Amoxicillin, 4x1000mg, 7 days | - |
| Oral Candida | 0.46% | 2.40% | 1.80% | 0% | - | Fluconazole, 1x100mg, 7 days | - |
| Genital Ulcers | 0.31% | 1.40% | 1.10% | 0% | - | Doxycycline, 2x100mg, 7 days | Rapid plasma reagin (RPR) |
| Esophageal Candida | 0.00% | 0.15% | 0.15% | 20-30% | 5-7 days | Fluconazole, 1x200mg, 7-14 days | - |
| Extra Pulmonary TB | 0.15% | 0.69% | 0.38% | 90-100% | 14-21 days | RHZE 2 months, RHZ 10 months | Chest X-ray, 3 AFB smears |
| Cryptococcal Meningitis | 0.08% | 0.23% | 0.08% | 100% | 21-28 days | Amphotericin B, 2x50mg, 14 days; Fluconazole, 1x400mg, 14-365 days | Antigen test, lumbar puncture |
| Kaposi's Sarcoma- Cutaneous | 0.00% | 0.23% | 0.23% | 20-40% | 7 days | Start ART | - |
| Herpes Simplex | 0.08% | 0.46% | 0.46% | 0% | - | Aciclovir, 3x400mg, 5-10 days | - |
| Kaposi's Sarcoma- Visceral | 0.00% | 0.08% | 0.00% | 80-90% | 14-28 days | Start ART | - |
| Urethritis | 0.08% | 0.08% | 0.08% | 0% | - | Doxycycline, 2x100mg, 7 days | - |
| *Based on data from Macha | | | | | | | |
| **Based on expert opinion of three physicians, one in Macha, two from Erasmus MC | | | | | | | |
