# Supplementary material for: Cost-Effectiveness of Pre-Exposure Prophylaxis (PrEP) in Preventing HIV-1 Infections in Rural Zambia: A Modeling Study
Source: PLoS One. 2013 Mar 18;8(3):e59549. doi: 10.1371/journal.pone.0059549 (PMC3601101; doi:10.1371/journal.pone.0059549)
Supplement: Text S1 — Model Description and equations. (DOC) [file pone.0059549.s007.doc]

**Text S1: Model Description and equations**

The state variables and HIV transmission equations for the model are shown below. There are four activity classes *i* based on the partner acquisition rate change: class 1 in which individuals have 7-31 partners per year, class 2 with 1.5-2.6 partners, class 3 with 0.1 and class 4 with 0.03.

The model included four HIV infection stages *k*: class 1 is the acute stage, class 2 is the chronic stage, class 3 is the pre-final AIDS stage in which individuals have limited sexual activity. Class 4 is the final AIDS stage in which patients do not have any sexual intercourse [1].

During treatment, the model includes two infection stages *l*: class 1 are individuals who were in the recent or chronic stage before start of treatment, class 2 are patients who were in one of the AIDS stage before antiretroviral therapy was initiated.

A proportion of individuals can be assigned to receive pre-exposure prophylaxis (PrEP) or not (
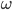
). Of those that are assigned PrEP, a proportion will develop resistance due to PrEP (
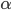
). The model includes two states of drug resistance *r*, state 1 is infected with a non-resistant virus, state 2 has a resistant virus due to PrEP use.

Patients progress through three treatment stages *m*: The first two treatment stages occur, respectively, during the first three months (stage 1) and months four to six after start of treatment (stage 2). Patients receiving antiretrovirals for more than six months are in stage 3.

**State variables**

$E_{i}$ = Entry rate susceptible individuals, i=1..4

$S_{i}$ = Susceptible individuals, i=1..4

$S_{i}^{P}$ = Susceptible individuals on PrEP, i=1..4

$I_{i,k,r}^{U}$ = HIV infected individuals, unaware of their infection, i=1..4, k=1..4, r=1..2

$I_{i,k,r}^{T}$ = HIV infected individuals who tested positive for HIV, i=1..4, k=1..4, r=1..2

$I_{i,l,m}^{Rx}$ = Infected individuals receiving treatment, i=1..4, l=1..2, m=1..3

**Other variables**

$\lambda_{i,k}$ = Force of infection, i=1..4, k=1..4

$\lambda_{i,k}^{P}$ = Force of infection, with use of PrEP, i=1..4, k=1..4

$N_{i}$ = Number of individuals in sexual activity class i, i=1..4

$\mu$ = Mortality general population

$\mu_{k}$ = Mortality untreated HIV infected patients in infection stage *k,* k=1..4

$\mu_{l,m}^{Rx}$ = Mortality treated patients in treatment stages *l* and *m,* l=1..3, m=1,2

$\gamma_{k}$ = HIV infection progression rate by stage k, k=1..4

$\tau_{k}$ = Proportion of patients tested for HIV in stage k, k=1..4

$RX_{k}$ = Proportion of patients starting treatment in progression stage k, k=1..4

$\rho$ = Proportion of patients that are retained in care

$\alpha$ = Rate of development of HIV-resistant virus due to PrEP use

$\omega$ = Proportion of patients on PrEP

$d$ = Rate of discontinuation of antiretroviral treatment

= Effectiveness of PrEP

$\psi$ = Reduction in transmissibility of breakthrough infection

**Ordinary Differential Equations**

(1) $E_{i}=90,000-S_{i}-S_{i}^{P}-I_{i,k,r}^{U}-I_{i,k,r}^{T}-I_{l,m}^{Rx}$

(2) $S_{i}=E_{i}(1-\omega)-S_{i}\sum_{k=1}^{4} \lambda_{i,k}-S_{i}\mu$

(3) $S_{i}^{P}=E_{i}(\omega)-S_{i}^{P}\sum_{k=1}^{4} \lambda_{i,k}^{P}-S_{i}^{P}\mu$

(4) $I_{i,1,1}^{U}=S_{i}\sum_{k=1}^{4} \lambda_{i,k}+S_{i}^{P}(\sum_{k=1}^{4} \lambda_{i,k}^{P})(1-\alpha)-I_{i,1,1}^{U}(\mu_{1}+\gamma_{1}+\tau_{1})$

(5) $I_{i,1,2}^{U}=S_{i}^{P}(\sum_{k=1}^{4} \lambda_{i,k}^{P})\alpha-I_{i,1,2}^{U}(\mu_{1}+\gamma_{1}+\tau_{1})$

(6) $\begin{matrix} I_{i,k,r}^{U}=\gamma_{k-1}I_{i,k-1,r}^{U}-I_{i,k,r}^{U}(\mu_{k}+\gamma_{k}+\tau_{k}) \\ k=2,3 \end{matrix}$

(7) $I_{i,4,r}^{U}=\gamma_{3}I_{i,3,r}^{U}-I_{i,4,r}^{U}(\mu_{4}+\tau_{4})$

(8)$I_{i,1,r}^{T}=I_{i,1,r}^{U}\tau_{1}-I_{i,1,r}^{T}(\mu_{1}+\gamma_{1}+\rho RX_{1})$

(9)$\begin{matrix} I_{i,k,r}^{T}=I_{i,k,r}^{U}\tau_{k}+\gamma_{k-1}I_{i,k-1,r}^{T}-I_{i,k,r}^{T}(\mu_{k}+\gamma_{k}+\rho RX_{k}) \\ k=2,3 \end{matrix}$

(10) $I_{i,4,r}^{T}=I_{i,4,r}^{U}\tau_{4}+\gamma_{3}I_{i,3,r}^{T}-I_{i,4,r}^{T}(\mu_{4}+\rho RX_{4})$

(11) $I_{i,1,1}^{Rx}=\rho(I_{i,1,r}^{T}*RX_{1}+I_{i,2,r}^{T}*RX_{2})-I_{i,1,1}^{Rx}(4+d+\mu_{1,1}^{Rx})$

(12) $I_{i,2,1}^{Rx}=\rho(I_{i,3,r}^{T}RX_{3}+I_{i,4,r}^{T}RX_{4})-I_{i,2,1}^{Rx}(4+d+\mu_{2,1}^{Rx})$

(13)$I_{i,l,2}^{Rx}=4I_{i,l,1}^{Rx}-I_{i,l,2}^{Rx}(4+d+\mu_{l,2}^{Rx})$

(14)$I_{i,l,3}^{Rx}=4I_{i,l,2}^{Rx}-I_{i,l,3}^{Rx}(d+\mu_{l,3}^{Rx})$

**Force of infection**

The equation for the force of infection includes a mixing matrix $M_{i,k}^{U}$ for patients unaware of their infection and a matrix $M_{i,k}^{T}$ for patients aware of their infection, with a different infectiousness for each stage, $\beta_{i,k}$ . The elements of this matrix are *i,k* and represent the probability that an individual with *i* new partnerships per year will form a new partnership with a member who has *k* new partners. The rate at which the sexual partner changes for individuals in each sexual activity group *i* is expressed as $c_{i}$. The values of the matrix depend on the degree of mixing ε. This degree can be fully assortative (ε=1), where partnerships are only formed within the same activity class. Or fully random (ε=0), where partnerships are randomly formed between different activity classes [2].

(15)$M_{i,k}^{U}=\varepsilon\delta+\frac{(1-\varepsilon)c_{k}N_{k}}{\sum_{i=1}^{4} c_{i}N_{i}}$

(16)$M_{i,k}^{T}=\varepsilon\delta+\frac{(1-\varepsilon)(1-\zeta)c_{k}N_{k}}{\sum_{i=1}^{4} (1-\zeta)c_{i}N_{i}}$

Where δ = 1 when *i = k*, and δ = 0 when *i ≠ k*. Furthermore, ζ is the proportional reduction in acquisition of new partnerships after patients become aware of their infection. ζ ranges between 0 and 40% for k=1,2 and ζ = 0 for k=3,4.

$$\lambda_{i,k}=\lambda_{i,k}^{U}+\lambda_{i,k}^{T}+\lambda_{i,k}^{RX}$$

$$\lambda_{i,1}^{U}=(1-\psi)c_{i}\frac{M_{i,1}^{U}}{N_{1}}(\beta_{i,1}I_{i,1}^{U})$$

$$\begin{matrix} \lambda_{i,k}^{U}=c_{i}\sum_{k=2}^{4} \frac{M_{i,k}^{U}}{N_{k}}(\beta_{i,k}I_{i,k}^{U}) \\ k=2,3,4 \end{matrix}$$

$$\lambda_{i,1}^{T}=(1-\psi)(1-\zeta)c_{i}\frac{M_{i,1}^{T}}{N_{1}}(\beta_{i,1}I_{i,1}^{T})$$

$$\begin{matrix} \lambda_{i,k}^{T}=(1-\zeta)c_{i}\sum_{k=2}^{4} \frac{M_{i,k}^{T}}{N_{k}}(\beta_{i,k}I_{i,k}^{T}) \\ k=2,3,4 \end{matrix}$$

$$\lambda_{i,k}^{RX}=(1-\zeta)c_{i}\sum_{k=1}^{4} \frac{M_{i,k}^{T}}{N_{k}}(\beta_{i,k}I_{l,m}^{RX})$$

In which $\lambda_{i,k}^{U}$is the force of infection due to contact with patients unaware of their infection. Similarly, and $\lambda_{i,k}^{RX}$are the forces of infection due to contacts with patients tested positive for HIV and patients receiving treatment. Additionally, $\lambda_{i,k}^{P}$is the force of infection due to contact of people on PrEP with all infected patients.

1. Hollingsworth TD, Anderson RM, Fraser C (2008) HIV-1 transmission, by stage of infection. J Infect Dis 198: 687-693.

2. Garnett GP, Anderson RM (1993) Factors controlling the spread of HIV in heterosexual communities in developing countries: patterns of mixing between different age and sexual activity classes. Philos Trans R Soc Lond B Biol Sci 342: 137-159.
